# Domain Adaptation-Based Sorting Method for UAV Swarm Targets on Multi-Station Features

**DOI:** 10.3390/s26144343

**Published:** 2026-07-08

**Authors:** Xihui Zhang, Meng Zhang, Wen Sun, Yinuo Ji, Ruihan Chen, Tao Liu

**Affiliations:** 1 Southwest China Institute of Electronic Technology, Chengdu 610036, China; seaharm_yeah@163.com (X.Z.); mengzhang@cqu.edu.cn (M.Z.); sw84114505@126.com (W.S.); 2 School of Microelectronics and Communication Engineering, Chongqing University, Chongqing 400044, China; 20193984@cqu.edu.cn (Y.J.); ruihanchen@stu.cqu.edu.cn (R.C.)

**Keywords:** UAV swarm sorting, hopping instant feature, domain adaptation

## Abstract

Existing target sorting methods suffer severe performance degradation or even failure under inherent severe spectrum overlap, homogeneous protocol parameters, and scarce single-source points in Synchronous Non-Orthogonal Frequency Hopping (SNOFH) scenarios. To address this challenge, this paper proposes a passive sorting framework for SNOFH UAV swarm signals based on multi-station relative hopping time difference. The proposed framework constructs a spatial-location-driven sorting feature system, designs a kernel joint distribution adaptation module to eliminate inter-station measurement discrepancies, and develops a multi-scale wavelet-based method to achieve sub-sampling level hopping time extraction, reducing the dependence on prior FH parameters and hardware radio frequency fingerprints. Experimental comparisons between the proposed and reference sorting methods are conducted on a simulated SNOFH dataset to validate the performance of the proposed sorting framework. The experimental results show that the proposed method achieves the highest sorting accuracy of 98%, outperforming adopted baselines in most SNOFH cases. The proposed method exhibits favorable robustness with noise interference, clock-synchronization error, carrier-frequency offset and multipath influence. It is a suitable choice for UAV swarm sorting under regular and slow-varying UAV formations.

## 1. Introduction

With the rapid development of collaboration methods of multiple Unmanned Aerial Vehicles (UAVs), swarm drones have been widely applied in various fields, including coordinated strike [1], battlefield reconnaissance [2], and security monitoring [3]. However, the growing proliferation of swarm drones has made an anti-swarm demand for several civil and military scenarios [4,5]. Multi-target sorting determines the upper limit of situational awareness performance of the entire anti-swarm system [6], establishing the critical correlation between received signals and individual UAV identities across consecutive observation instants. Among current multi-target sorting approaches, the electromagnetic signal-based one has become the core pathway to counter malicious swarm threats, with inherent advantages of all-weather operation, wide-area coverage, and anti-occlusion capability [7,8,9]. Moreover, the Synchronous Non-Orthogonal Frequency Hopping (SNOFH), a frequency-hopping (FH) communication scenario characterized by time synchronization and spectrum overlapping, becomes an important UAV communication manner that simultaneously balances the spectrum utilization efficiency, anti-interception performance, and concurrent communication efficiency [10,11]. Hence, sorting SNOFH swarm drones based on electromagnetic signals turns out to be an attractive issue in anti-swarm research.

Existing mainstream electromagnetic detection architectures are broadly divided into two well-established categories: active radar detection and passive radio frequency (RF) sensing. The active radar detection often suffers from high exposure risk, large power consumption, and vulnerability to electronic countermeasures [12,13,14]. Conversely, the passive RF detection mainly focusing on FH signals has become an attractive issue in anti-swarm countermeasures, owing to its strong concealment, low cost, and flexible deployment [15,16].

Three technical routes are involved in passive RF sorting. The first is the radio frequency fingerprint (RFF)-based approach, which extracts unique hardware features from received RF signals to distinguish individual UAV emitters and complete cross-frame data association. Soltani et al. [17] proposed a multi-classifier framework consisting of 1D deep networks on RFFs; Ezuma et al. [18,19] conducted single UAV detection and classification using RFF of the signals transmitted from the controller; Huynh-The et al. [20] developed a particular convolutional network structure to effectively detect and classify drones, and recognize associated operations. The second is the underdetermined blind source separation (UBSS)-based method, which aims to recover individual source signals from overlapped received mixtures using single-source point (SSP) samples when the number of receiving antennas is less than the number of swarm targets. Li et al. [21] designed an adaptive denoising and azimuth difference single-source detection method to improve mixing matrix estimation accuracy in an UBSS process; Fu et al. [22] proposed a UBSS scheme for synchronous FH signal sorting without any spectrum overlapping; Zhang et al. [23] constructed a third-order covariance tensor and realized mixing matrix estimation, while effectively relaxing the sparsity limitation and improving target separation efficiency and accuracy. The third category is the hopping parameter feature-based method, which commonly sorts targets by extracting and matching protocol-level FH parameters, including hopping time, hopping period, FH set, and dwell time. Zhu et al. [24] incorporated energy-based features into protocol-level FH parameters for fixed and sweep frequency interference counteraction.

Recently, learning-based UAV detection algorithms have attracted growing attention from researchers. The superiority of such methods lies in their task-oriented mining of data features, which can eliminate biases introduced by manual experience in feature engineering. Deep network architectures, including convolutional neural networks [25] and long short-term memory networks [26], have been widely adopted for UAV detection. Wang et al. [27] proposed a data-driven context-level FH signal parameter extractor to gain blind FH parameter estimation for target sorting. Lin et al. [28] developed a YOLO-based model for time-frequency parameter estimation and sorted targets in a uniform circular array. Nevertheless, these approaches inherently suffer from the common drawbacks of learning-based algorithms: massive labeled data is required for network training [29], making them poorly suited for UAV detection scenarios characterized by few-shot or even zero-shot training. Although existing works have alleviated this conflict via the “pre-training and fine-tuning” paradigm [30], they still fail to fundamentally address the challenges encountered by learning-based methods in real-time feature separation and matching of multiple UAV targets.

All mentioned technical routes face inherent challenges in the sorting of SNOFH UAV swarm signals. RFF-based methods rely on pure single-source signals to extract discriminative hardware imperfection fingerprints, and thus suffer severe performance degradation or even complete failure under frequency-domain aliasing of concurrent multi-target signals [18]. UBSS-based schemes require abundant effective SSPs for accurate mixing matrix estimation, while the extreme scarcity of native SSPs in SNOFH scenarios leads to fatal estimation bias of the mixing matrix and invalid source reconstruction [31,32]. For hopping parameter-based methods, the highly homogeneous protocol-level hopping parameters of SNOFH swarms submerge the subtle inter-node differences in aliasing and noise, making it impossible to achieve reliable individual UAV discrimination and cross-slot association [33]. Although learning-based methods can realize end-to-end processing in form, they still exhibit insufficient intelligence at the signal processing level and demand massive labeled data, resulting in prominent limitations.

To address the aforementioned inherent limitations of existing multi-target sorting methods in SNOFH scenarios, we utilize the truth that the spatial distance between two UAVs determines their relative signal propagation delay arriving at a certain reconnaissance station. Accordingly, we propose a multi-station sorting method for SNOFH swarm signals, relying on the different UAVs ’ signal propagation delays, instead of hardware-level RFF and protocol-level hopping features, collected from different reconnaissance stations. It achieves robust target sorting throughout continuous movement of the UAV swarm. The proposed framework consists of four phases, including target number estimation, hopping instant feature abstraction, domain adaptation-based feature drift compensation, and data sorting between stations. We utilize multi-scale continuous wavelet transform (CWT) and cubic spline interpolation to suppress spectral aliasing and acquire sub-sampling-precision hopping edge timestamps for hopping instant feature abstraction, and deploy kernel joint distribution adaptation (KJDA) for intelligent signal processing to counteract feature drift stemming from UAV swarm motion. Distinct from existing hopping time estimation methods, this integrated scheme elevates the precision of target hopping time features and supports UAV swarm sorting using features distorted by temporal and spatial propagation drift. In addition, we extend the domain adaptation paradigm to individual UAV discrimination. The key innovations and contributions of this work are summarized as follows:

(1) A SNOFH multi-target sorting method using multi-station hopping instant features, free of protocol-level FH parameters and RF fingerprints.
(2) Nonlinear domain adaptation suppressing the drift of multi-station signal delays for stable UAV Identity (ID) sorting.
(3) A high-precision UAV hopping time estimation under severe spectrum overlap.

The remainder of this paper is organized as follows: Section 2 elaborates on the proposed multi-target sorting framework, including feature design and core module principles. Section 3 illustrates the simulated SNOFH data used for performance evaluation. Section 4 validates the method’s performance through comparative experiments with the SNOFH signal data. Finally, Section 5 concludes the paper and discusses future research directions.

## 2. Methods

### 2.1. Notations

**Figure 1:**
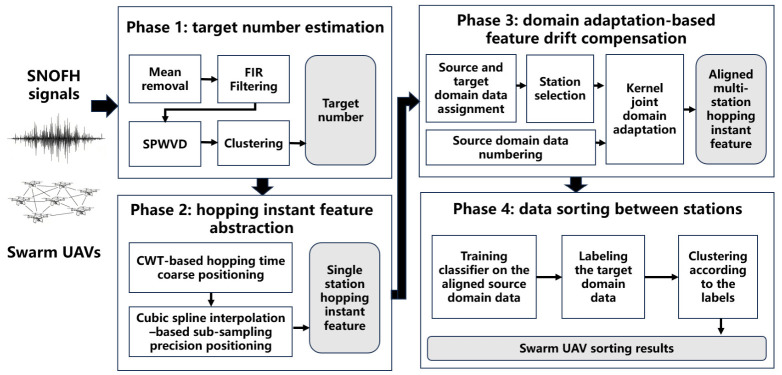
Overall workflow of the proposed four-phase passive sorting framework.

In this paper, we consider a four-phase passive sorting framework, consisting of M distributed ground surveillance stations, which synchronously receive SNOFH signals transmitted by N UAV swarm targets. Figure 1 shows the overall workflow of the proposed method. The receiver of each station adopts a uniform sampling rate $F_{s}$, with the sampling period defined as $T_{s}=1/F_{s}$. For the *m*-th monitoring station where $m\in \left\{1,2,\ldots ,M\right\}$, the received discrete baseband signal $X_{m}[n]$ is denoted as follows:

(1) $$X_{m}[n]=\sum_{i=1}^{N}h_{m,i}\cdot s_{i}(n-T_{s}\tau_{m,i})+w_{m}[n]$$

where n=0,1,…,L−1 is the discrete time index, L is the total length of the sampled signal, $h_{m,i}$ is the complex channel gain between the *i*-th UAV and the m-th station, $s_{i}\left(\cdot \right)$ is the normalized FH baseband signal transmitted by the i-th UAV, $\tau_{m,i}$ is the free-space propagation delay of the signal, and $w_{m}[n]$ is the Additive White Gaussian Noise (AWGN) with zero mean and variance $\sigma_{w}^{2}$.

We denote the Smoothed Pseudo Wigner-Ville Distribution (SPWVD) for time-frequency analysis as SPWVD(n,f), where f is the normalized frequency variable. For CWT, the wavelet coefficient sequence is denoted as W(a,n), with a as the scale parameter controlling the time-frequency resolution. The relative time-difference vector of the m-th monitoring station is denoted as $\tau_{m}$, and the multi-station joint feature vector of the i-th UAV target is denoted as $f_{i}$ where $i\in \left\{1,2,\ldots ,N\right\}$. For domain adaptation, we define the feature dataset of the initial sorting period with pseudo labels as the source domain $D_{s}$, and the feature dataset of the current period to be sorted as the target domain $D_{t}$.

### 2.2. Phase 1: Target Number Estimation

In this subsection, we eliminate out-of-band interference and extract aliasing-free SSPs to predict the number of targets and support subsequent feature extraction.

For the received signal $X_{m}[n]$, we first perform mean removal and an FIR bandpass as follows:

(2) $$X_{m,\mathrm{pre}}[n]=\left(X_{m}[n]-\frac{1}{L}\sum_{k=0}^{L-1}X_{m}[k]\right)*b[n]$$

where $b\left[n\right]$ is the impulse response of the FIR filter with passband matched to the prior frequency range of the SNOFH signal, and * denotes the discrete convolution operator. Then, we adopt SPWVD to generate the time-frequency energy spectrum, which suppresses cross-term interference in multi-source aliased scenarios while maintaining high time-frequency resolution [34]. The discrete form of SPWVD is:

(3) $${\mathrm{SPWVD}}_{m}(n,f)=\sum_{p=-P}^{P}g[p]\sum_{q=-Q}^{Q}h[q]\cdot X_{m,\mathrm{pre}}[n+p+q]X_{m,\mathrm{pre}}^{*}[n+p-q]e^{-j4\pi fqT_{s}}$$

where $g\left[\cdot \right]$ and $h\left[\cdot \right]$ are time-domain and frequency-domain Gaussian smoothing windows, P and Q are the half-lengths of the smoothing windows, and ${\left(\cdot \right)}^{*}$ denotes the complex conjugate operator.

We detect SSPs based on the subspace orthogonality criterion [31]. For each time-frequency point, we construct the time-frequency observation vector $z\left(n,f\right)={\mathrm{SPWVD}}_{m}\left(n,f\right)$, and perform eigenvalue decomposition on its autocorrelation matrix by:

(4) $$R_{zz}=E\{z(n,f)z^{H}(n,f)\}=U_{s}\Lambda_{s}U_{s}^{H}+U_{n}\Lambda_{n}U_{n}^{H}$$

where $U_{s}$ and $U_{n}$ are the signal subspace and noise subspace, respectively, $\Lambda_{s}$ and $\Lambda_{n}$ are the diagonal matrices of corresponding eigenvalues, and ${\left(\cdot \right)}^{H}$ denotes the conjugate transpose operator. After that, a time-frequency point can be judged as a valid SSP if it satisfies the condition $\sum_{k=1}^{K}\lambda_{k}/\lambda \lambda_{max}$, where $\lambda_{max}$ is the maximum eigenvalue of $R_{zz}$, $\theta_{\lambda }$ is a preset threshold and K is the total number of eigenvalues. According to the extracted SSPs, we can determine energy-concentrated regions from the time-frequency spectrum and use Density Peak Clustering (DPC) [35,36] to estimate the number of UAV targets $\hat{N}$.

### 2.3. Phase 2: Hopping Instant Feature Abstraction

In this subsection, we integrate multi-scale CWT and cubic spline interpolation to abstract high-precision features representing the spatial position for an individual UAV.

Considering that CWT can separate overlapping spectra and owns a continuous scale adjustment range [37], we choose multi-scale CWT decomposition to detect the FH signal transient edge based on the time-domain signal $X_{SSP}\left[n\right]$ corresponding to each extracted SSP. We select the first-order Gaussian derivative wavelet as the mother wavelet. Accordingly, the CWT $X_{SSP}\left[n\right]$ can be calculated as:

(5) $$W(a,n)=\frac{1}{a}\sum_{k=0}^{L_{\mathrm{SSP}}-1}X_{\mathrm{SSP}}[k]\cdot \psi^{*}(a(k-n)T_{s})$$

where $\psi \left(\cdot \right)$ is the CWT function, $L_{SSP}$ is the sample length of the SSP signal, and a is the scale parameter. In this study, we use both small-scale and large-scale parameters to obtain high time resolution and strong anti-noise performance. We select local modulus maxima of the wavelet coefficients at the used scale parameters if a corresponding maximum within ±Δn sampling points at all larger scales exists (we set Δn=3 in this study). Thus, the peak position corresponding to the rising edge of the FH signal is the coarse positioning integer sampling point $n_{\mathrm{peak}}$. To break through the accuracy limitation of discrete sampling, we use cubic spline interpolation [27,28] to achieve sub-sampling-precision positioning of the hopping time $t_{\mathrm{fine}}$.

To construct features reflecting the distance between individual UAVs and *M*-stations, we take the earliest detected hopping instant in the same dwell period as the reference $t_{\mathrm{ref},m}=\min\left(t_{\mathrm{fine},m,1},\ldots ,t_{\mathrm{fine},m,\hat{N}}\right)$ and calculate the relative time difference for each target:

(6) $$\tau_{m,i}=t_{\mathrm{fine},m,i}-t_{\mathrm{ref},m},\;i=1,2,\ldots ,\hat{N}$$

Thus, the relative time-difference vector of the m-th station can be expressed as:

(7) $$\begin{array}{cc} {\bar{\tau }}_{m} & =\left[{\bar{\tau }}_{m,1},{\bar{\tau }}_{m,2},\ldots ,{\bar{\tau }}_{m,\hat{N}}\right]\\ & ={\left[\tau_{m,1},\tau_{m,2},\ldots ,\tau_{m,\hat{N}}\right]}^{T}/\sqrt{\sum_{i=1}^{\hat{N}}\tau_{m,i}^{2}} \end{array}$$

This feature is uniquely determined by the normalized spatial position of the UAVs in the swarm, which is not affected by the homogenization of protocol-level FH parameters [38].

### 2.4. Phase 3: Domain Adaptation-Based Feature Drift Compensation

In this subsection, we attempt to solve the nonlinear drift effect $\tau_{m}$ calculated from different stations via domain adaptation, a learning paradigm reducing the distribution discrepancy between source and target domains with unlabeled target data. Domain adaptation solves the station-sensitive feature alignment problem, fully utilizing the swarm drone formation and individual relative spatial position. In theory, the inter-station deviation of the relative time-difference vector is governed by the geometric layout of monitoring stations and UAV spatial positions. Nevertheless, the proposed sorting framework is designed as a pre-processing module for subsequent passive target geolocation, where the real geometric coordinates of UAVs are completely unknown and cannot be acquired in advance during the sorting stage. Thus, a closed-form geometric transformation would require prior UAV position information, leading to an inherent logical dilemma: accurate geometry is required to calibrate time-difference features, whereas calibrated time-difference features are exactly the fundamental input for solving UAV geometry. For this reason, we resort to domain adaptation to solve the nonlinear drift of $\tau_{m}$ across stations. It performs data-driven nonlinear alignment purely based on the statistical distribution of interference-distorted multi-station features, without relying on any prior UAV geometric information, which conforms to the inherent logical order of passive sorting-then-positioning tasks.

Firstly, we select an arbitrary station as the standard one, and define the feature dataset from the standard one as the source-domain $D_{s}={\left\{{\bar{\tau }}_{m,i}\right\}}_{i=1}^{\hat{N}}{|}_{m=m\_std}$, where m_std denotes the serial number of the standard station. The feature dataset from the other station to be sorted is defined as the target domain $D_{t}={\left\{{\bar{\tau }}_{m,i}\right\}}_{i=1}^{\hat{N}}{|}_{m\neq m\_std}$. We label the target IDs in the source-domain samples as:

(8) $$D_{s}={\left\{({\bar{\tau }}_{m,i},y_{i})\right\}}_{i=1}^{N_{s}}{|}_{m=m\_std}$$

where $y_{i}\in \{1,2,\ldots ,\hat{N}\}$ is the pseudo label of the i-th feature, and $N_{s}=\hat{N}$ is the number of source-domain samples. Here, the pseudo-label assigned to each source-domain sample is randomly generated. The pseudo labels serve as initial identifiers for the source-domain samples, and do not rely on any prior information. The maximum value $N_{s}$ of such identifiers is determined by the number of hopping instants $\hat{N}$ extracted in Phase 2. Considering *M* stations, we can collect the feature samples from *M*-1 non-standard stations and combine them as the target domain. Meanwhile, the source-domain dataset is solely constructed from relative hopping time-difference features collected by one standard monitoring station. The total number of pseudo-label categories is equal to the estimated number of UAV targets. Random permutation of these label numbers only changes the digital identifier of each feature cluster, while the intrinsic cluster partition structure of source-domain samples remains fixed. The optimization objective of KJDA only requires the relative distribution gap between different feature categories, rather than the specific numerical value of each category label. Regardless of how the exclusive pseudo-labels are randomly allocated, the solved optimal projection matrix for feature alignment keeps consistent, which guarantees deterministic cross-station clustering and sorting outputs without random fluctuations. As a result, the randomly assigned pseudo-labels will not bring randomness to the cross-station sorting results after KJDA alignment.

Secondly, we attempt to align both the marginal and conditional distributions of the source and target domains with KJDA, which extends the linear joint distribution adaptation (JDA) framework to nonlinear scenarios via the kernel trick for low-resource systems [39]. We primarily establish the original feature matrix **X** as follows:

(9) $$X=\left[{\bar{\tau }}_{s},{\bar{\tau }}_{t}\right]=\left[{\bar{\tau }}_{m=m\_std},{\bar{\tau }}_{t}\right]=\left[x_{1},\ldots ,x_{M}\right]\in {\mathbb{R}}^{N_{s}\times M}$$

where ${\bar{\tau }}_{s}\in {\mathbb{R}}^{N_{s}\times 1}$ and ${\bar{\tau }}_{t}\in {\mathbb{R}}^{N_{s}\times (M-1)}$ represent the source and target domain original feature matrices, respectively. Considering special scenarios including symmetrical formations, close-range targets and geometric degradation, the relative hopping time differences extracted from a single reconnaissance station for different targets may become extremely small and degrade the sorting performance. Accordingly, we require that the total number of deployed reconnaissance stations is more than 4. Then, we set *M* = 4 and select the top 4 stations with the highest quality of relative hopping time differences from all geographically dispersed reconnaissance stations for subsequent processing. The selection criterion here is the variance of feature vectors: a larger variance indicates better discriminability and higher signal quality. Then, we map the original features to a high-dimensional Reproducing Kernel Hilbert Space (RKHS) via a nonlinear kernel mapping $\phi \left(\cdot \right)$ to construct the combined RKHS feature matrix **K** as follows:

(10) $$K=\left\{K_{ij}\right\}=\left\{\phi \left(x_{i},x_{j}\right)\right\}=\left\{\exp\left(\frac{{\left\|x_{i}-x_{j}\right\|}_{2}}{\sigma^{2}}\right)\right\}\in {\mathbb{R}}^{N_{s}\times N_{s}}$$

We construct the marginal distribution alignment matrix $M_{0}$ and the conditional distribution alignment matrix $M_{c}$ for each class *y_i_*:

(11) $$M_{0}=\frac{1}{N_{s}^{2}}1_{s}1_{s}^{T}+\frac{1}{N_{t}^{2}}1_{t}1_{t}^{T}-\frac{1}{N_{s}N_{t}}1_{s}1_{t}^{T}-\frac{1}{N_{s}N_{t}}1_{t}1_{s}^{T}$$

(12) $$M_{c}=\frac{1}{N_{s,c}^{2}}1_{s,c}1_{s,c}^{T}+\frac{1}{N_{t,c}^{2}}1_{t,c}1_{t,c}^{T}-\frac{1}{N_{s,c}N_{t,c}}1_{s,c}1_{t,c}^{T}-\frac{1}{N_{s,c}N_{t,c}}1_{t,c}1_{s,c}^{T}$$

where $1_{s}$ and $1_{t}$ are indicator vectors for the source and target domains, respectively. $N_{s,c}$ and $N_{t,c}$ are the number of samples of pseudo-label c in the source and target domains, respectively. $1_{s,c}$ and $1_{t,c}$ are the corresponding class indicator vectors. Thus, we can establish a learning model to find the optimal projection matrix A in the RKHS, which minimizes the distribution difference between domains while maximizing the feature discriminability:

(13) $$\begin{array}{l} \underset{A}{\min}\mathrm{tr}(A^{T}KMK^{T}A)+\lambda {\left\|A\right\|}_{F}^{2}\\ s.t.\;A^{T}KHK^{T}A=I \end{array}$$

where λ is the regularization coefficient, $M=M_{0}+\sum_{c=1}^{\hat{N}}M_{c}$ is the total distribution alignment matrix, $H=I-\left(1/N_{s}+N_{t}\right)1\cdot 1^{T}$ is the centering matrix, and $\mathrm{tr}\left(\cdot \right)$ is the matrix trace operator. Formula (11) is an optimization problem that can be solved via generalized eigenvalue decomposition, and the optimal projection matrix $A^{*}$ is obtained from the smallest m generalized eigenvectors. Finally, the aligned feature matrix can be computed by:

(14) $$Z={\left(A^{*}\right)}^{T}K$$

where Z is the calibrated sample matrix involving all the feature samples of the standard and non-standard stations, eliminating the spatial effect caused by different station positions.

### 2.5. Phase 4: Data Sorting Between Stations

We conduct final target sorting and ID assignment to ensure sorting precision and robustness on multi-station hopping instant features.

We train an ID classifier $f_{\mathrm{ID}}\left(\cdot \right)$ based on the standard station data from the calibrated sample matrix Z with associated IDs. Then, we use the learned $f_{\mathrm{ID}}\left(\cdot \right)$ to classify the remaining projected data in Z. The classification results will guide data clustering across the multiple stations. We assume that the projected hopping instant features with an identical label are from the same UAV.

The final output of the proposed framework includes the fixed ID, high-precision hopping instant sequence, and multi-station feature vector of each UAV target, which supports subsequent passive positioning and tracking.

The whole process of the proposed method is shown in Algorithm 1.
**Algorithm 1**. Pseudo-code of the proposed cross-station feature sorting method**Input**: Multi-station received SNOFH signal, sampling rate, sliding window length **Output**: estimated target number, matched hopping instant feature matrix  // Phase 1: Target Number Estimation  1: Preprocess: Mean removal + FIR bandpass filtering according to Formula (2)  2: Compute SPWVD to obtain time-frequency energy spectrum based on Formula (3)  3: Detect SSPs via Formula (4)  4: Extract time-frequency energy concentration regions  5: Estimate target number $\hat{N}$ via DPC on SSPs  // Phase 2: Hopping Instant Feature Abstraction  6: for each detected SSP signal do  7:        Perform multi-scale CWT with scale a1, a2 according to Formula (5)  8:        Locate wavelet modulus maxima to obtain coarse hopping instant  9:        Refine sub-sampling instant via cubic spline interpolation  10: end for  11: Compute relative time-difference feature based on Formula (6)  12: Construct a relative time-difference feature vector according to Formula (7)  // Phase 3: KJDA-based Feature Alignment  13: Calculate feature vector variance for each reconnaissance station  14: Select the top 4 stations with the largest variance  15: Constitute source-domain (standard station) and target domain via Formula (8)  16: Assign random pseudo labels to source-domain samples  17: Construct kernel matrix K via RBF kernel according to Formulas (9) and (10)  18: Obtain aligned feature matrix **Z** in the projected subspace based on Formulas (11)–(14)  // Phase 4: Cross-Station Sorting  19: Train ID classifier on aligned source-domain features  20: Classify target domain features and assign a consistent UAV ID  21: Output estimated target number $\hat{N}$, and matched hopping instant feature matrix **Z**

## 3. Dataset Formulation

We have built an SNOFH dataset to validate the proposed multi-target sorting framework. All data are generated following the signal model defined in Section 2.1, with full consideration of practical UAV swarm motion, formation variation, and spectrum overlap characteristics in real-world scenarios. The proposed framework relies on two preset experimental assumptions: perfect unified timing references among all reconnaissance stations and a fixed, known geographic deployment of each station. In practical engineering, nanosecond-level synchronous timing can be realized by equipping each receiver with global navigation satellite system disciplined high-precision atomic clock modules, which provide the required consistent timing benchmark for extracting relative hopping time-difference features. Meanwhile, fixed station layout is a widely adopted deployment mode for ground permanent anti-UAV reconnaissance systems, whose coordinate information can be pre-measured via surveying equipment before operation. Hence, in subsequent parts, we assume all stations maintain nanosecond-level synchronization and hold fixed positions.

### 3.1. Monitoring Station Deployment

**Figure 2:**
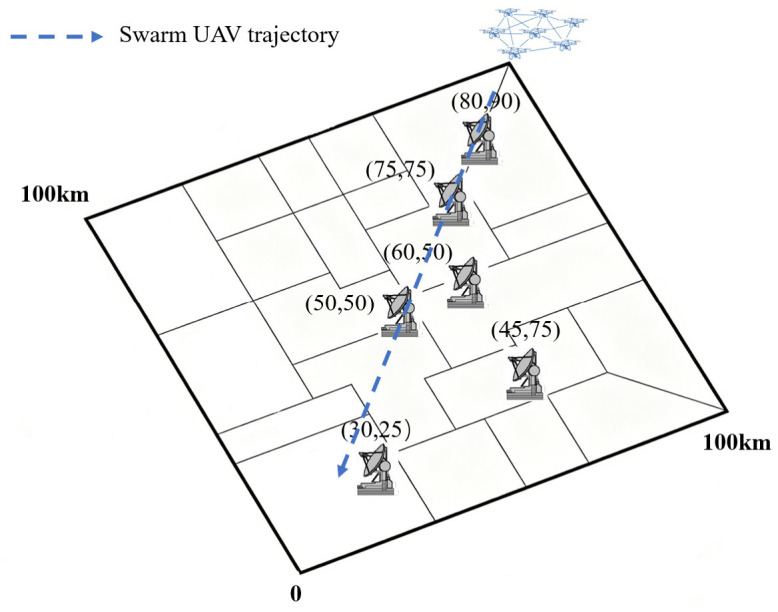
Deployment of the six ground monitoring stations in the simulated planar monitoring area.

The dataset contains simulated SNOFH signal samples received by six fixed ground monitoring stations, deployed within a 100 km × 100 km planar monitoring area. All stations are set at an altitude of 0 m. The planar coordinates (in km) of the six stations are (30, 25), (45, 75), (50, 50), (60, 50), (75, 75) and (80, 90), as illustrated in Figure 2.

### 3.2. UAV Swarm Motion and Formation Configuration

**Figure 3:**
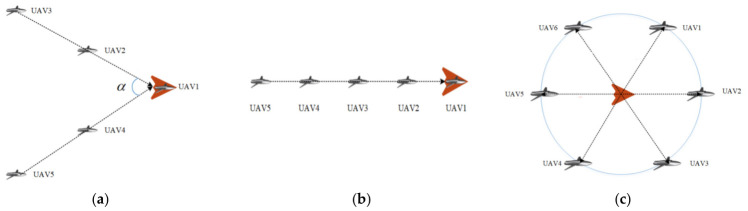
UAV swarm formation patterns: (**a**) broken-line formation, (**b**) straight-line formation, and (**c**) circular formation.

The simulated swarm consists of 10 UAVs, with a minimum inter-UAV flight distance of 50 m and a constant flight altitude of 200 m. The geometric center of the swarm moves at a constant speed of 15 m/s, traveling from the initial coordinate (90, 90) km to the end point (25, 25) km. The total flight duration is approximately 204 min. Three typical formation patterns are set during the flight, as shown in Figure 3. The broken-line, straight-line, and circular formations are denoted as Formation A, Formation B, and Formation C, respectively. The time division of these formations is: Formation A is adopted from the 0th to the 150th minute; Formation B occupies 151st to the 180th minute; Formation C dominates the period of the 181st to 204th minute.

### 3.3. SNOFH Signal Configuration

**Figure 4:**
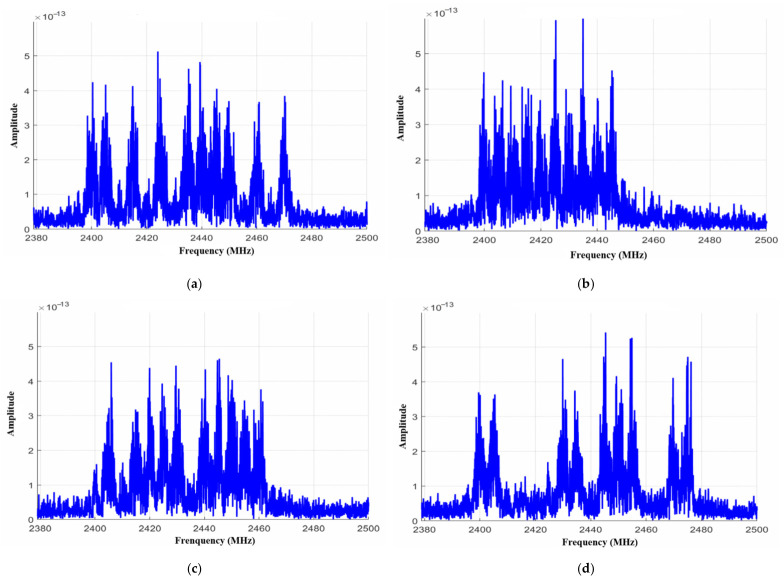
Frequency—main waveforms of the generated SNOFH swarm signal in (**a**) hop slot 1, (**b**) hop slot 2, (**c**) hop slot 3, and (**d**) hop slot 4.

All UAVs in the swarm adopt the SNOFH communication scheme throughout the flight. The FH pattern is generated by Gold codes, with a hopping rate of 2000 hops per second. The operating frequency range is 2.4 GHz to 2.475 GHz, containing 16 hopping frequency points evenly spaced at 5 MHz intervals. We set a unique bit rate of 40 Mbps for each UAV, and adopt random binary bits to simulate the information to be transmitted. We use 16 QAM modulation for communication. Thus, the symbol rate of each UAV transmitter can be calculated by 40M/log16=10M (Baud/s). With a raised-cosine pulse shaping filter of roll-off factor *a* = 0.5, the instantaneous RF bandwidth occupied by a single FH channel is $10M\;\;\times \left(0.5+1\right)/2=7.5\;\;(\mathrm{MHz})$. Under this configuration, the signal spectra of two adjacent FH channels overlap by 50%. On this basis, we can generate radio frequency superposition signals of UAV swarms with varying degrees of spectral overlap by selecting different roll-off factors. Finally, we can superpose the individual signals of all UAVs to obtain the composite signal illustrated. The basic frequency-domain electromagnetic signal waveforms of the generated SNOFH swarm communication signal with Signal to Noise Ratio (SNR) = 0 dB, station-level clock-synchronization error $\sigma_{clk}$ = 0 ns, carrier-frequency offset Δf = 0 Hz, and no multipath interference are shown in Figure 4a–d.

To verify the robustness and identify the failure boundary of the proposed method, we can add AWGN to the basic electromagnetic signals to obtain a typical SNR range from −10 dB to 15 dB with an interval of 5 dB, covering low-SNR harsh electromagnetic environments and ideal SNR scenarios. We artificially introduce controlled delays to delay the hopping time obtained from a reconnaissance station to expand the maximum clock-synchronization error from 0 ns to 5 ns, 10 ns, 20 ns, and 50 ns. We also randomly select 10% UAVs to apply an extra carrier-frequency offset ranging from 0 Hz to 20 kHz, representing the frequency mismatch and phase rotation effect caused by oscillator inaccuracies between the UAV transmitter and the station receiver. We simulate multipath effects by introducing amplitude attenuation and phase delay based on the basic electromagnetic signals. Accordingly, the multipath effect is characterized by the multipath amplitude attenuation coefficient and multipath relative time delay. We select four multipath frequency-domain parameter groups: amplitude attenuation 0.5 and delay 50 ns, amplitude attenuation 0.4 and delay 100 ns, attenuation 0.3 and delay 150 ns, and amplitude attenuation 0.2 and delay 200 ns, to demonstrate the impact of multipath interference from weak to strong.

## 4. Experimental Validation

### 4.1. Experimental Settings

#### 4.1.1. Evaluation Metrics

We adopt two sorting metrics calculated for independent single-dwell-slot results: Target Number Estimation Accuracy (TNEA) and Sorting Accuracy (SA).

TNEA evaluates the performance of the proposed method in estimating the total number of UAV targets. It calculates the proportion of test cases across M stations where the estimated target number is consistent with the ground-truth number, and quantitatively reflects the accuracy of target quantity estimation as follows:

(15) $$\mathrm{TNEA}=\frac{1}{M}\sum_{i=1}^{M}\frac{N_{est}}{N_{tar}}\times 100\%$$

where $N_{est}$ and $N_{tar}$ denote the estimated and real number of UAVs in a single dwell slot.

SA reflects the sorting correctness by the ratio of correctly clustered signal samples to the total number of samples. Its mathematical formula is as follows:

(16) $$\mathrm{SA}=\frac{1}{N}\sum_{i=1}^{N}Ι\left(\mathrm{cluster}\left(x_{i}\right)=\mathrm{label}\left(x_{i}\right)\right)$$

where *N* is the total number of signal samples, $x_{i}$ is the *i*-th sample, cluster(⋅) is the clustering result of the sorting method, label(⋅) is the ground-truth UAV label, and I(⋅) is the indicator function. I(⋅) outputs one when the condition is true, otherwise zero.

#### 4.1.2. Baseline Methods

Different from conventional FH-based sorting schemes, this study focuses on the SNOFH scenario and aims to achieve cross-station matching of swarm target hopping instant features, which makes it difficult to adopt a single existing method as a complete baseline. Therefore, we construct comparative baselines through a reasonable combination of mainstream technologies. Following the inherent processing pipeline of the proposed method, all baseline schemes are established under a unified framework consisting of three core procedures: hopping time extraction, feature transformation, and cross-station clustering, to realize inter-station target feature matching for systematic comparison.

Based on the mentioned processing pipeline, three reference methods (Baseline A, Baseline B, and Baseline C) are constructed. At the hopping time extraction stage:

- Baseline A adopts the signal separation and hopping instant estimation method proposed in Ref. [21], which separates single-source signals based on adaptive sparse reconstruction and global maximum likelihood estimation.
- Baseline B utilizes the time-frequency energy mutation and sparsity characteristics of hopping signals to determine hopping time features, following the technical scheme used in Ref. [23].
- Baseline C employs the local contrast measure (LCM) strategy introduced in Ref. [40] to search for hopping time parameters from time-frequency feature maps.

Notably, we only extract and reproduce the hopping time parameter extraction core module from each cited reference instead of adopting their complete original end-to-end models. All model parameters and threshold settings for hopping instant estimation strictly follow the recommended configurations provided in the corresponding references. The number of effective hopping instants detected by each baseline method is regarded as the estimated number of UAV targets, which provides a data-driven basis for subsequent clustering tasks.

To ensure consistent and fair comparison conditions across all baseline methods, a unified feature transformation and cross-station clustering strategy is adopted in the subsequent processing stages. First, the hopping time differences obtained from the previous stage are converted into unified formation topology vectors according to Formulas (9) and (10). Then, we use the Constant False Alarm Rate(CFAR) K-means clustering algorithm proposed in Ref. [41] as the cross-station matching part for all baseline schemes. We set the CFAR threshold *ε* = 0.08 and the maximum iteration number = 300. The CFAR K-means is a specific sorting method for trajectory measurement with excellent adaptability to changing data. It optimizes the initial cluster seed selection by utilizing the CFAR threshold of feature distribution, and introduces a clustering iteration termination criterion constrained by feature discrimination loss. We believe these two improvements can suppress clustering deviation caused by aliased hopping features and time-delay feature drift, making the clustering process adaptive to SNOFH signals. The cluster number fed into CFAR K-means is adaptively assigned as the target number estimated by each baseline’s own hopping instant extraction module, without injecting ground-truth target count prior to maintain unsupervised experimental conditions.

#### 4.1.3. Scenario and Method Setup

We divide five formation modes from the dataset to verify the robustness of the proposed method under various UAV swarm formations:

(1) Mode A: Full data of Formation A (broken line, 0–150 min)
(2) Mode B: Full data of Formation B (straight line, 151–180 min)
(3) Mode C: Full data of Formation C (circular, 181–204 min)
(4) Mode A → B: Data of the formation transition period from A to B (145–155 min)
(5) Mode B → C: Data of the formation transition period from B to C (175–185 min)

For the proposed method, we set the CWT scale parameter a=2 and a=15 for ultra-high resolution hopping edge positioning and anti-noise edge screening, respectively. We use an RBF kernel for KJDA with σ = 1.0, and set KJDA regularization coefficient λ = 0.01. For domain adaptation, we gather the hopping time-difference feature data of 10 consecutive hopping dwell slots of the standard station as the source domain, and the ones of all non-standard stations as the target domain.

All simulation configurations, including monitoring station coordinates, UAV flight trajectory parameters, SNOFH waveform parameters, noise intensity, clock-synchronization error, carrier-frequency offset and multipath attenuation settings are fully provided in Table 1. The algorithm hyperparameters of the proposed method, such as CWT scale parameters, RBF kernel width, KJDA regularization coefficient, and source-domain sample length, are also fixed and listed in Table 1.

All experimental results in Section 4.2, Section 4.3, Section 4.4 and Section 4.5 are averaged over 20 independent Monte Carlo runs. Each run generates a new independent SNOFH dataset adopting different Gold code segments to produce distinct FH patterns. This ensures sufficient statistical diversity of the simulated communication signals. Each test run contains 1000 independent dwell slots. We report the mean value and standard deviation of TNEA and SA, and the corresponding 95% confidence intervals are implied in the statistical fluctuation.

### 4.2. Model Parameter Sensitivity Analysis

Multiple hyperparameters dominate the feature extraction, domain alignment and cross-station sorting performance of the proposed framework. In this subsection, we carry out model parameter sensitivity experiments to quantify how each core parameter affects sorting performance and adopt SA as the primary evaluation metric. During each group of tests, only the target parameter is modified, and all remaining hyperparameters are locked to the unified baseline configuration. The baseline values are set as: *a*_1_ = 2, *a*_2_ = 15, regularization coefficient *λ* = 0.1, RBF kernel width *σ* = 1.0, source-domain window size T_s_ = 10 dwell slots.

#### 4.2.1. Sensitivity Analysis of Small CWT Scale

**Figure 5:**
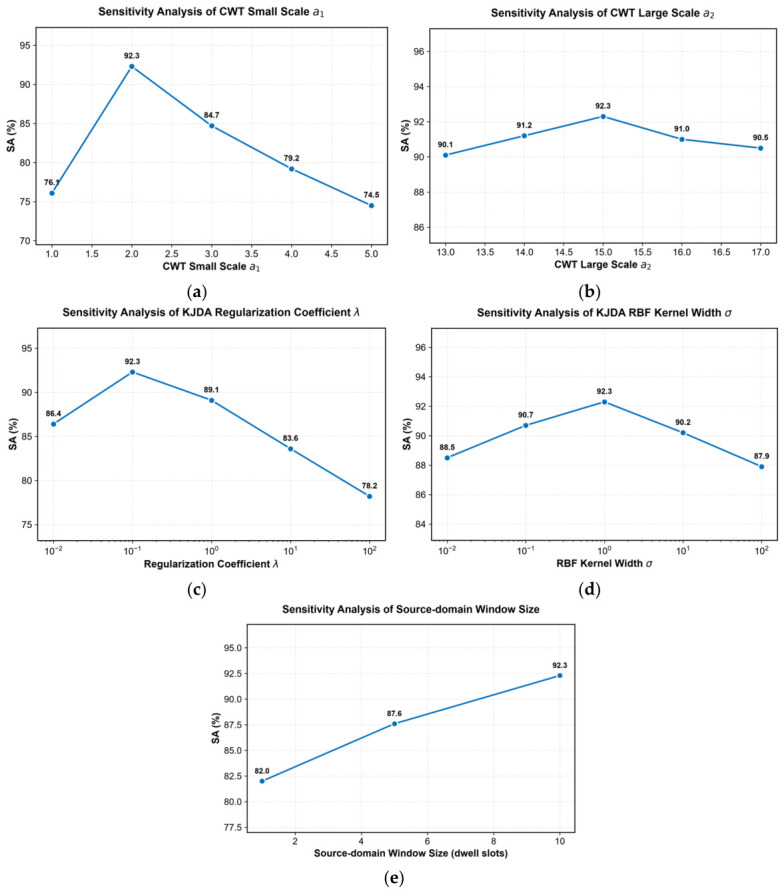
Performance of proposed framework with different parameter settings: (**a**) CWT small-scale parameter, (**b**) CWT large-scale parameter, (**c**) KJDA regularization coefficient, (**d**) KJDA kernel width, and (**e**) source-domain window size.

We set *a*_1_ ∈ {1, 2, 3, 4, 5} and fix *a*_2_ = 15, *λ* = 0.1, *σ* = 1.0, and T_s_ = 10. The SA results under different *a*_1_ are listed in Figure 5a. The peak SA reaches 92.3% at *a*_1_ = 2, which corresponds to the baseline configuration adopted throughout this study.

The sorting performance drops rapidly when *a*_1_ deviates from two. The small-scale *a*_1_ is designed to capture precise hopping time instants of individual UAVs. The test results demonstrate that *a*_1_ = 2 perfectly matches the time scale of dense electromagnetic energy jumps of UAV swarms. The sharp performance degradation indicates that the accurate extraction of hopping time features is highly sensitive to the setting of *a*_1_. If *a*_1_ is too small, the wavelet lacks sufficient time-frequency resolution to suppress ambient noise; if *a*_1_ is excessively large, tiny hopping edge information is smoothed out, introducing severe timing bias for subsequent time-difference feature construction.

#### 4.2.2. Sensitivity Analysis of Large CWT Scale

We set *a*_2_ ∈ {13, 14, 15, 16, 17} and *a*_1_ = 2, *λ* = 0.1, *σ* = 1.0, and T_s_ = 10. The SA results under different *a*_2_ are summarized in Figure 5b.

The optimal performance is achieved at *a*_2_ = 15. Unlike *a*_1_, performance only slightly and uniformly declines within the range [13,17]. The large-scale *a*_2_ adapts to the time scale of signal dwell slots, extracting stable electromagnetic energy accumulated within each dwell duration. Within [13,17], we observe weak sensitivity of SA to minor adjustments of *a*_2_, because the corresponding time scale can sufficiently aggregate signal energy near each carrier frequency without causing mutual interference of energy from multiple adjacent dwell slots.

#### 4.2.3. Sensitivity Analysis of KJDA Regularization Coefficient

We set *λ* ∈ {0.01, 0.1, 1, 10, 100} and fix *a*_1_ = 2, *a*_2_ = 15, *σ* = 1.0, and T_s_ = 10. The SA results under different *λ* are shown in Figure 5c.

The maximum SA appears at *λ* = 0.1. We found obvious fluctuating performance loss when *λ* deviates from the optimal value. In fact, the regularization coefficient *λ* controls the generalization capacity of cross-station domain alignment. When *λ* is too small, the generalization constraint is insufficient, the model adopts an over-fit clustering granularity and overemphasizes trivial fluctuations of feature differences, which cannot accommodate the dynamic motion of UAV swarms. In contrast, an overlarge *λ* may excessively weaken the weight of feature variation, blurring the inter-UAV feature gaps and leading to degraded cross-station matching accuracy. Thus, the KJDA regularization coefficient is an important parameter that should be determined by either experience or prior knowledge.

#### 4.2.4. Sensitivity Analysis of KJDA Kernel Width

We set *σ* ∈ {0.01, 0.1, 1, 10, 100} and fix *a*_1_ = 2, *a*_2_ = 15, *λ* = 0.1, and T_s_ = 10. The SA results under different *σ* are shown in Figure 5d.

The optimal kernel width is *σ* = 1 with the highest SA. The performance presents oscillating downward trends as *σ* moves away from one, yet the overall degradation amplitude remains limited. This phenomenon verifies that the proposed hopping time-difference features can effectively characterize swarm formation structures. The intrinsic discriminability embedded in spatial time-delay features makes the whole sorting algorithm robust against moderate changes in *σ*. A tiny *σ* magnifies irrelevant noise-induced feature deviations, while an overly large *σ* weakens the distinguishing power between different UAV clusters; neither leads to a catastrophic performance drop in this feature system.

#### 4.2.5. Sensitivity Analysis of Source-Domain Window Size

We test three source-domain window sizes T_s_ in 1, 5 and 10 consecutive dwell slots, with all other parameters fixed to baseline values and the target-domain window size unchanged. The SA results of varied T_s_ are illustrated in Figure 5e.

We find that the sorting performance improves monotonically with the increase in source-domain window size, and the peak SA is obtained at T_s_ = 10. We infer that two factors jointly account for this trend. First, a longer source-domain window collects richer feature samples from the standard monitoring station, providing more sufficient category prior information to guide feature matching between source and target domains in KJDA projection. Second, a window containing 10 dwell slots only covers a total time span of 5 ms, considering 2000 hops/s. Within such a short period, the formation topology of UAV swarms and their relative positions to monitoring stations barely change, so feature samples belonging to the same UAV maintain high similarity, which guarantees reliable pseudo-label division and stable domain alignment. If the window is too short (one or five dwell slots), insufficient feature samples lead to ambiguous cluster boundaries and lower cross-station sorting accuracy.

### 4.3. Performance Under Varied Interference

#### 4.3.1. SNR Variation

This section evaluates the performance of all methods under SNR ranging from −10 dB to 15 dB with a 5 dB step. Other dataset parameters settings are: spectrum overlap 50%, clock-synchronization error 0 ns, carrier-frequency offset 0 Hz, and no multipath effect.

From Table 2, it can be observed that both TNEA and SA of all methods gradually increase as the SNR rises, which conforms to the inherent logic that accurate target number estimation (high TNEA) is a necessary prerequisite for satisfactory SA.

It can be seen that Baseline A and Baseline C show obvious performance limitations at low SNR. For instance, at SNR = −10 dB, Baseline A only achieves TNEA of 0.71 and SA of 42.8%, while Baseline C merely obtains TNEA = 0.58 and SA = 27.1%. Benefiting from multi-scale CWT and cubic spline interpolation, the proposed method acquires a TNEA of 0.82 and SA of 70.5% at −10 dB, which is far higher than all comparison methods. When the SNR increases to 15 dB, the TNEA of the proposed method reaches 0.98 with SA up to 98.0%, maintaining a clear performance advantage over Baseline B with TNEA = 0.88, SA = 79.6%.

In terms of environmental adaptability, Baseline C is extremely sensitive to low-SNR fading, and its TNEA is always the lowest across all SNR conditions. By contrast, Baseline A and B have moderate anti-noise capability but cannot effectively suppress the feature ambiguity caused by spectrum overlap, leading to saturated SA below 80% even at high SNR. The proposed method exhibits strong adaptability under medium and high SNR conditions, but its performance also degrades obviously when SNR drops below −5 dB. At −10 dB, the advantage in SA is narrowed to around 28% compared with Baseline A, rather than maintaining an overwhelming gap.

This reveals the performance boundary of the proposed method: it is highly applicable and reliable for SNOFH sorting under moderate and high SNR (≥0 dB). In extremely low-SNR scenarios below −5 dB, noise severely distorts time-frequency hopping edges, limiting the upper bound of TNEA and further restraining the sorting performance improvement.

#### 4.3.2. Clock-Synchronization Error Variation

We compare the performance of all methods under clock-synchronization error ranging from 0 ns to 50 ns. Other dataset parameters settings are: SNR = 0 dB, spectrum overlap 50%, carrier-frequency offset 0 Hz, and no multipath effect.

As shown in Table 3, the case of 0 ns clock error is completely consistent with the baseline result at SNR = 0 dB, which guarantees experimental consistency under ideal clock synchronization. With the increase in clock drift, both TNEA and SA of all methods degrade monotonically.

Within a 10 ns clock error, the proposed method still retains TNEA above 0.90 and SA over 86.8%, far outperforming Baseline A (0.74 and 43.5%) and Baseline C (0.61 and 44.3%). When clock drift reaches 50 ns, the proposed method declines to TNEA 0.77 and SA 72.8%, and the performance gap with UBSS baselines is greatly narrowed.

Baseline A and B maintain relatively stable TNEA but suffer from persistent poor SA, as they cannot calibrate the time-difference ambiguity induced by clock-synchronization error. Baseline C is most sensitive to clock deviation; its time-frequency contrast is easily distorted, leading to the lowest TNEA and SA at all error levels.

In summary, the proposed method maintains stable sorting performance when the clock-synchronization error is no more than 10 ns; once the clock drift exceeds 20 ns, the sub-sampling hopping time deviation exceeds the compensation capacity of KJDA, resulting in severe SA degradation. This constitutes an obvious inherent limitation of our framework under large timing drift.

#### 4.3.3. Different Carrier-Frequency Offset

We evaluate the performance of all methods with varied carrier-frequency offsets from 0 to 20 kHz. Other dataset parameters settings are: SNR = 0 dB, spectrum overlap 50%, clock-synchronization error 0 ns, and no multipath effect.

Table 4 keeps the zero-offset case fully consistent with the SNR = 0 dB baseline in Table 2, ensuring experimental uniformity under ideal carrier-frequency conditions. As carrier-frequency offset increases, time-frequency spectrum distortion and phase rotation become more severe, causing continuous drops in TNEA and SA for all methods.

Under a small offset within 10 kHz, the proposed method still maintains TNEA above 0.87 and SA over 83.5%, which is substantially higher than all baselines. When the offset rises to 20 kHz, the proposed method degrades to TNEA 0.74, and SA 68.3%, and the performance advantage is obviously compressed.

Baseline A and B show mild degradation in target number estimation, but cannot compensate for offset-induced feature drift, leading to mediocre sorting performance. Baseline C is severely affected by spectrum aliasing, always yielding the worst TNEA and SA.

The proposed algorithm works reliably when the carrier-frequency offset is controlled within 10 kHz. If the offset exceeds 15 kHz, severe time-frequency spectrum distortion cannot be fully eliminated by multi-scale CWT, which forms a clear performance bottleneck and limits the practical deployment of this method under large oscillator frequency mismatch.

#### 4.3.4. Multipath Interference Variation

In this section, we adopt a two-path propagation model for all UAV swarm signals. Four multipath groups are defined with different attenuation coefficients and relative time delays.

- Group 1 (mild multipath): Main path + secondary path with amplitude attenuation 0.5 and delay 50 ns
- Group 2 (moderate multipath): Main path + secondary path with amplitude attenuation 0.4 and delay 100 ns
- Group 3 (severe multipath): Main path + secondary path with amplitude attenuation 0.3 and delay 150 ns
- Group 4 (severe multipath): Main path + secondary path with amplitude attenuation 0.2 and delay 200 ns

Other dataset parameters settings are: SNR = 0 dB, spectrum overlap 50%, clock-synchronization error 0 ns, and carrier-frequency offset 0 Hz.

As shown in Table 5, all results under two-path multipath interference are lower than the baseline performance at SNR = 0 dB in Table 2, which is physically reasonable since multipath superposition inevitably aggravates spectrum aliasing and hopping feature distortion.

In Group 1, the proposed method still maintains clear superiority, with TNEA = 0.92 and SA = 89.5%. At this weak interference level, the multi-scale CWT and cubic spline interpolation can effectively separate real hopping edges from low-amplitude short-delay multipath components, thus keeping accurate target number estimation and stable sorting performance.

When entering Group 2, the proposed method suffers a sharp performance drop. The TNEA of the proposed method decreases drastically from 0.92 to 0.76, leading to a significant SA decline from 89.5% to 72.1%. The essential reason is that the medium delay and moderate attenuation induce complicated spectrum aliasing, which seriously interferes with the proposed method’s ability to discriminate the number of hopping instants. The dramatic degradation of TNEA further drags down the subsequent SA, forming an obvious performance inflection point at Group 2.

In Groups 3 and 4, the delays, including 150 and 200 ns, are almost equivalent to the maximum time difference of 167 ns obtained by dividing the maximum spacing of drones (50 m) by the speed of light. Thus, the performance of the proposed method continues to deteriorate and gradually approaches or even slightly lags behind Baseline A and Baseline B. The core mechanism is twofold: (1) severe multipath blurs the separability of hopping instant features, making the proposed method unable to accurately distinguish individual UAV hopping events, which further reduces TNEA; and (2) the proposed sorting framework heavily relies on the distinguishable relative time-difference features. Strong multipath destroys the inherent spatial–temporal feature uniqueness, causing inevitable errors in hopping instant estimation. Such dual deterioration on both target number estimation and feature separability makes the proposed method nearly ineffective under strong multipath conditions.

In contrast, Baseline A and B, based on UBSS, are less sensitive to spectrum aliasing caused by multipath. Although their overall SA is not high, their TNEA remains relatively stable and does not collapse sharply with the increase in multipath delay. Baseline C always performs the worst due to its vulnerability to time-frequency contrast degradation.

The proposed framework only achieves robust sorting under weak multipath interference with relative delay ≤50 ns. When multipath delay reaches or exceeds 100 ns, multipath components blur the unique spatial time-difference features relied on by our sorting scheme, leading to a sharp drop in TNEA and SA. Under severe multipath with delay above 150 ns, the proposed method even underperforms UBSS-based baselines, which is a critical application limitation of our feature-driven architecture.

#### 4.3.5. Different Formation Modes

We set the dataset parameters as SNR = 0 dB, spectrum overlap 50%, clock-synchronization error 0 ns, carrier-frequency offset 0 Hz, and no multipath effect for formation robustness.

Table 6 shows that all methods achieve much better TNEA and SA under regular formation modes (A, B, C) than formation transition modes (A → B, B → C). For all algorithms, low TNEA always corresponds to low SA, which further validates that target number estimation is the essential precondition of subsequent sorting.

Under regular circular Formation C, the proposed method obtains TNEA = 0.96 and SA = 92.7%, while Baseline C only achieves SA of 57.0%. In contrast, under rapid formation transition A → B, the proposed method’s TNEA drops sharply to 0.72, and SA falls to 63.5%, and its overall performance is only slightly better than Baselines A and B, with no overwhelming advantage.

Baseline A and B have stable but non-excellent TNEA in all formation scenarios; their SA cannot exceed 62% even in the most regular formation, due to the inability to adapt to spatial time-difference variation. Baseline C always presents the lowest TNEA and SA, especially in transition modes where TNEA drops around 0.52. The proposed method has excellent adaptability to regular and slowly varying formations, but its domain adaptation module relies on the static spatial distribution assumption, which is severely violated during rapid formation switching.

The proposed method is designed for regular and slowly varying UAV swarm formations. When the swarm undergoes a rapid structural transition, the static spatial distribution assumption required by KJDA domain adaptation is violated, which drastically reduces cross-station feature alignment accuracy and weakens the overall sorting performance. This limits the applicability of our method for highly dynamic drone swarms with frequent formation switching.

#### 4.3.6. Different Spectrum Overlap Levels

To investigate the influence of spectrum aliasing on sorting performance, we set the maximum spectrum overlap between adjacent frequency points as 10%, 20%, 30%, 40%, and 50%. All experiments are conducted at SNR = 0 dB, without clock-synchronization error, carrier-frequency offset, and multipath interference.

As shown in Table 7, with the decrease in spectrum overlap, the time-frequency aliasing degree is gradually alleviated, so the TNEA and SA of all methods increase monotonically. The proposed method also shows a clear rising trend, achieving the best performance at the spectrum overlap of 50% and continuously improving as the overlap decreases.

At a high spectrum overlap of 50%, severe time-frequency aliasing seriously blurs hopping edge features. Baseline A and B only reach TNEA of 0.69 and 0.70, while Baseline C is only 0.58. Benefiting from multi-scale CWT and cubic spline interpolation, the proposed method still maintains a high TNEA of 0.90 and SA of 86.5%, showing obvious superiority over all comparison methods.

As the spectrum overlap decreases from 50% to 20%, the TNEA advantage of the proposed method gradually shrinks. The reason is that reduced spectrum overlap makes time-frequency energy contours clearer, enabling Baseline A, B, and C to identify hopping instants more accurately and continuously improve TNEA. However, the SA gap between the proposed method and the baselines does not narrow significantly. Even at 20% overlap, the SA of Baseline A and B are only 77.8% and 79.3%, far lower than the proposed method’s 92.7%. This is because the baseline methods lack the kernel domain adaptation module and cannot eliminate inherent inter-station feature drift. Even if they can estimate the target number accurately, the inter-station feature mismatch still leads to poor sorting performance.

When the spectrum overlap drops to 10%, the time-frequency aliasing is almost negligible. All methods achieve high estimation accuracy, with the TNEA of Baseline A and B reaching 0.97, and the proposed method only rising to 0.95. Meanwhile, the SA of all methods increases to around 91–94%, and the proposed method cannot achieve the top performance in a slight gap. In this low-overlay scenario, the multi-scale anti-aliasing advantage of the proposed method is no longer prominent. The baseline methods can already complete an accurate target number estimation, and the inter-station feature mismatch is greatly weakened, making the KJDA alignment advantage less obvious. Thus, the performance of the proposed method lags slightly behind that of the reference methods.

The results on different spectrum overlaps indicate that the proposed method has prominent performance advantages under moderate to high spectrum overlap (30–50%), and can effectively suppress time-frequency aliasing to guarantee both TNEA and SA. When the spectrum overlap is lower than 20%, the advantage of the proposed method gradually converges. At a low overlap of 10%, all methods achieve near-saturated accuracy, and the proposed method cannot maintain superiority, which defines the applicable scope and inherent performance limit of the proposed sorting framework.

### 4.4. Performance Under Different Spectrum Occupancy Levels

To investigate how swarm signal density affects sorting performance, we define Spectrum Occupancy Level (SOL) as the ratio of the number of UAVs *N* to the number of hopping channels *N_hc_* by

(17) $$\mathrm{SOL}=N/N_{hc}$$

A larger SOL indicates higher frequency resource utilization and more severe time-domain overlap among concurrent hopping signals. In this section, we fix the total number of hopping channels at 16 and vary the UAV swarm size from 2 to 10 with a step of two, yielding SOLs from 2/16 to 10/16. All experiments are conducted under interference-free conditions using the optimal parameter configuration of the proposed method. SA and TNEA are adopted as evaluation metrics, and each test is repeated over 20 Monte Carlo runs.

As shown in Table 8, both SA and TNEA exhibit a consistent downward trend as the SOL increases from 2/16 to 10/16. When only one UAV is present (occupancy = 2/16), the proposed method achieves nearly perfect sorting performance with SA close to 100% and TNEA equal to 1.0, since there is no inter-signal overlap and the hopping time features can be extracted with extremely high precision. As the number of UAVs gradually increases, the frequency channels are occupied more densely, and the performance degrades steadily. At the maximum tested occupancy of 10/16, the method still maintains SA = 92.3 ± 0.9% and TNEA = 0.95 ± 0.01, demonstrating acceptable robustness for moderately dense swarm scenarios.

According to the definition of SOL, a higher SOL value means more UAVs transmit simultaneously within the same frequency band, which makes the rising edges of signal energy more crowded in the time domain. The mutual interference among adjacent UAV hopping signals becomes more pronounced, and the multi-scale CWT module is more likely to be disturbed by overlapping energy from neighboring emitters when locating hopping instants. As a result, the extracted hopping time features suffer from larger estimation errors, and the subsequent relative time-difference features used for sorting suffer more severe distortion, which ultimately leads to the observed decline in both SA and TNEA. This trend is consistent with the physical intuition that denser signal superposition increases the difficulty of blind sorting.

### 4.5. Ablation Test

We conduct an ablation test to verify the effectiveness of each core module of the proposed method. All experiments are conducted under the fixed condition of SNR = 0 dB, Formation Mode C, with 1000 independent dwell slots tested. Based on the proposed full model, we formulate two sub-models: one is without the CWT part, and the other lacks the KJDA process.

We utilize Short-Time Fourier Transform (STFT) to replace CWT, considering the necessity of time-frequency transformation in hopping instant feature abstraction. In Table 9, we discover 11.1% and 0.20 drops in SA and TNEA, respectively, by replacing CWT with STFT. This is because STFT has a fixed time-frequency resolution, which cannot balance high time resolution for transient edges and strong anti-noise performance. In contrast, multi-scale CWT can adaptively adjust the time-frequency resolution, accurately capturing the hopping edge even under spectrum overlap and noise interference. Hence, multi-scale CWT is the basis for obtaining high-precision core sorting features.

KJDA Domain Alignment is more critical than multi-scale CWT, according to the performance drops in Table 5. We observe 15.5% drop of SA and a 0.218 drop of TNEA when KJDA is missing, which is more obvious than the ones of multi-scale CWT. We infer that the distribution of hopping instant feature measurements varies significantly across different stations due to the changeable relative distance between the UAVs and stations. Without KJDA-based inter-station feature alignment, the same UAV’s features from different stations are scattered in the feature space, leading to severe clustering errors. Thus, this module is the key to ensuring the compactness of same-UAV features and the separability of different-UAV features.

All core modules of the proposed method are necessary and complementary. The multi-scale CWT module ensures high-precision feature extraction, and the KJDA module enhances feature separability via inter-station alignment. The ablation results fully verify the rationality and effectiveness of the proposed method’s module design.

## 5. Conclusions

SNOFH is an important communication scheme for UAV swarms, balancing spectrum utilization, anti-interception performance, and concurrent transmission efficiency. However, existing sorting methods always face fundamental challenges in SNOFH scenarios: severe spectrum overlap, homogeneous protocol features, and scarce SSPs lead to dramatic performance degradation, restricting passive monitoring capability for malicious UAV swarms.

This study proposes a passive SNOFH sorting framework based on multi-station relative hopping time difference, with three core contributions. First, we construct a hopping-instant-feature-driven sorting feature system, eliminating the dependence on pre-known FH parameters and hardware RFF features. Second, we introduce KJDA to align cross-station feature distributions, reducing inter-station measurement discrepancies and improving feature separability. Third, we develop a hopping time extraction method combining multi-scale CWT and multi-station joint verification, achieving sub-sampling level edge detection under the spectrum overlap condition.

The proposed method achieves promising sorting performance in typical SNOFH scenarios. Under medium-to-high SNR (≥0 dB), small clock-synchronization error (≤10 ns), low carrier-frequency offset (≤10 kHz), weak multipath interference, and regular UAV formations, it attains a maximum TNEA of 0.98 and SA of 98.0%, consistently outperforming Baseline A, B and C. The core advantages stem from multi-scale CWT sub-sampling, hopping instant extraction and KJDA nonlinear feature alignment, which effectively suppresses spectrum aliasing and inter-station feature drift.

We notice that the proposed framework has several clear inherent limitations. First, a large clock-synchronization error over 20 ns will introduce a non-negligible hopping time bias that cannot be fully compensated by the KJDA module, leading to obvious performance decay. Second, a carrier-frequency offset larger than 15 kHz causes irreversible time-frequency spectrum distortion, weakening the feature extraction capability of multi-scale CWT. Third, strong multipath interference with relative delay exceeding 100 ns distorts the spatial propagation delay difference features serving as the core sorting basis; in such cases, the proposed method loses its superiority against UBSS-based baselines. Fourth, the scheme relies on the quasi-static spatial distribution assumption of UAV swarms. Frequent and rapid formation transitions break this premise, making domain adaptation fail to align cross-station features effectively. Fifth, the fixed station deployment assumption restricts the proposed method to stationary monitoring systems. If the reconnaissance stations move randomly or their coordinates cannot be acquired in advance, the propagation delay difference between UAVs and stations cannot be calculated, making our spatial feature construction invalid. All the above limitations restrict the practical deployment of our method to scenarios with nanosecond-level synchronous timing, small timing drift, low carrier-frequency mismatch, weak multipath fading, stable drone formation, and fixed station deployment.

Future work will focus on dynamic domain adaptation with kinematic constraints, sorting–tracking fusion framework, semi-physical verification, and multi-station selection rules.

## Figures and Tables

**Table 1:** Main experimental and parameter settings.

Parameter Category	Parameter Symbol	Value/Configuration
Monitoring Station	Deployment area	100 km × 100 km
	Station number	6 stations, select the top 4 for processing
	Altitude	0 m
UAV Swarm	UAV quantity	10
	Minimum inter-UAV distance	50 m
	Flight altitude	200 m
	Moving speed	15 m/s
	Formation mode	A (broken line), B (straight line), C (circular), A → B, B → C
SNOFH Signal	Hopping rate	2000 hops/s
	Frequency range	2.4–2.475 GHz
	Hopping points	16 points, 5 MHz interval
	Modulation	16 QAM
	Channel bandwidth	7.5 MHz
Interference	Spectrum overlap	0–50%
	SNR range	−10 dB~15 dB, step 5 dB
	Clock-synchronization error	0/5/10/20/50 ns
	Carrier-frequency offset	0/5/10/15/20 kHz
	Multipath setting	4 groups of attenuation and time-delay combinations
Proposed Method	CWT scale	*a*_1_ = 2, *a*_2_ = 15
	Mother wavelet	First-order Gaussian derivative
	KJDA kernel	RBF kernel, *σ* = 1.0
	Regularization coefficient	*λ* = 0.01
	Source-domain sample length	10 consecutive dwell slots
	Target number estimation	DPC over-extracted SSPs
Evaluation	Metrics	TNEA, SA

**Table 2:** TNEA and SA performance of all methods under different SNR levels.

SNR(dB)	Metric	Baseline A	Baseline B	Baseline C	Ours
−10	TNEA	0.71 ± 0.03	0.73 ± 0.02	0.58 ± 0.04	**0.82 ± 0.02**
	SA (%)	42.8 ± 1.7	44.5 ± 1.4	27.1 ± 2.0	**70.5 ± 1.5**
−5	TNEA	0.75 ± 0.02	0.76 ± 0.03	0.62 ± 0.03	**0.88 ± 0.02**
	SA (%)	51.2 ± 1.5	52.9 ± 1.2	35.0 ± 1.8	**79.3 ± 1.2**
0	TNEA	0.79 ± 0.02	0.80 ± 0.02	0.67 ± 0.03	**0.95 ± 0.01**
	SA (%)	56.8 ± 1.3	58.5 ± 1.1	53.7 ± 1.5	**92.3 ± 0.9**
5	TNEA	0.82 ± 0.03	0.83 ± 0.02	0.71 ± 0.02	**0.96 ± 0.01**
	SA (%)	67.9 ± 1.2	69.4 ± 1.0	65.3 ± 1.3	**95.6 ± 0.8**
10	TNEA	0.85 ± 0.02	0.86 ± 0.02	0.75 ± 0.03	**0.97 ± 0.01**
	SA (%)	75.0 ± 1.1	73.2 ± 0.9	71.8 ± 1.2	**97.1 ± 0.7**
15	TNEA	0.87 ± 0.02	0.88 ± 0.01	0.78 ± 0.02	**0.98 ± 0.01**
	SA (%)	79.6 ± 1.0	79.6 ± 0.8	76.9 ± 1.1	**98.0 ± 0.6**

**Table 3:** TNEA and SA under different clock-synchronization errors.

Clock Error	Metric	Baseline A	Baseline B	Baseline C	Ours
0 ns	TNEA	0.79 ± 0.02	0.80 ± 0.02	0.67 ± 0.03	**0.95 ± 0.01**
	SA (%)	56.8 ± 1.3	58.5 ± 1.1	53.7 ± 1.5	**92.3 ± 0.9**
5 ns	TNEA	0.77 ± 0.02	0.78 ± 0.02	0.64 ± 0.03	**0.93 ± 0.01**
	SA (%)	53.5 ± 1.3	55.1 ± 1.2	50.2 ± 1.5	**90.1 ± 0.9**
10 ns	TNEA	0.74 ± 0.03	0.75 ± 0.02	0.61 ± 0.04	**0.90 ± 0.02**
	SA (%)	43.5 ± 1.4	48.9 ± 1.4	44.3 ± 1.6	**8** **3** **.5 ± 1.1**
20 ns	TNEA	0.70 ± 0.03	0.71 ± 0.03	0.58 ± 0.04	**0.84 ± 0.02**
	SA (%)	45.3 ± 1.6	46.8 ± 1.4	41.4 ± 1.9	**80.5 ± 1.1**
50 ns	TNEA	0.65 ± 0.04	0.66 ± 0.03	0.54 ± 0.05	**0.77 ± 0.03**
	SA (%)	40.0 ± 1.8	41.4 ± 1.6	35.9 ± 2.1	**72.8 ± 1.3**

**Table 4:** TNEA and SA under different carrier-frequency offsets.

Carrier-Frequency Offset	Metric	Baseline A	Baseline B	Baseline C	Ours
0 kHz	TNEA	0.79 ± 0.02	0.80 ± 0.02	0.67 ± 0.03	**0.95 ± 0.01**
	SA (%)	56.8 ± 1.3	58.5 ± 1.1	53.7 ± 1.5	**92.3 ± 0.9**
5 kHz	TNEA	0.76 ± 0.02	0.77 ± 0.02	0.63 ± 0.03	**0.92 ± 0.01**
	SA (%)	53.4 ± 1.2	54.9 ± 1.1	49.6 ± 1.4	**89.2 ± 0.8**
10 kHz	TNEA	0.72 ± 0.03	0.73 ± 0.02	0.59 ± 0.04	**0.87 ± 0.02**
	SA (%)	48.7 ± 1.3	50.2 ± 1.2	44.5 ± 1.6	**83.5 ± 0.9**
15 kHz	TNEA	0.68 ± 0.03	0.69 ± 0.03	0.56 ± 0.04	**0.81 ± 0.02**
	SA (%)	44.1 ± 1.5	45.5 ± 1.3	39.8 ± 1.8	**76.4 ± 1.1**
20 kHz	TNEA	0.63 ± 0.04	0.64 ± 0.03	0.52 ± 0.05	**0.74 ± 0.03**
	SA (%)	38.8 ± 1.7	40.2 ± 1.5	35.1 ± 2.0	**68.3 ± 1.2**

**Table 5:** TNEA and SA under different multipath interference levels.

Multipath Group	Metric	Baseline A	Baseline B	Baseline C	Ours
Group 1 (0.5, 50 ns)	TNEA	0.77 ± 0.02	0.78 ± 0.02	0.65 ± 0.03	**0.92 ± 0.01**
	SA (%)	54.1 ± 1.2	55.7 ± 1.1	50.9 ± 1.4	**89.5 ± 0.8**
Group 2 (0.4, 100 ns)	TNEA	0.73 ± 0.02	0.74 ± 0.02	0.60 ± 0.03	**0.76 ± 0.03**
	SA (%)	49.0 ± 1.3	50.4 ± 1.2	46.2 ± 1.5	**72.1 ± 1.1**
Group 3 (0.3, 150 ns)	TNEA	0.69 ± 0.03	**0.70 ± 0.03**	0.55 ± 0.04	0.68 ± 0.03
	SA (%)	44.3 ± 1.4	**45.8 ± 1.3**	41.1 ± 1.7	42.1 ± 1.4
Group 4 (0.2, 200 ns)	TNEA	0.64 ± 0.03	**0.65 ± 0.03**	0.50 ± 0.04	0.62 ± 0.04
	SA (%)	40.1 ± 1.5	**41.3 ± 1.4**	36.0 ± 1.9	37.5 ± 1.6

**Table 6:** TNEA and SA under five formation modes.

Formation Mode	Metric	Baseline A	Baseline B	Baseline C	Ours
Mode A	TNEA	0.81 ± 0.02	0.82 ± 0.02	0.69 ± 0.03	**0.96 ± 0.01**
	SA (%)	53.7 ± 1.2	55.4 ± 1.1	50.7 ± 1.4	**92.3 ± 0.7**
Mode B	TNEA	0.82 ± 0.02	0.83 ± 0.02	0.70 ± 0.03	**0.95 ± 0.01**
	SA (%)	58.5 ± 1.1	60.2 ± 1.0	55.5 ± 1.3	**92.1 ± 0.8**
Mode C	TNEA	0.83 ± 0.01	0.84 ± 0.01	0.72 ± 0.02	**0.96 ± 0.01**
	SA (%)	60.0 ± 1.0	61.8 ± 0.9	57.0 ± 1.2	**92.7 ± 0.6**
A → B Transition	TNEA	0.65 ± 0.04	0.66 ± 0.03	0.52 ± 0.05	**0.72 ± 0.03**
	SA (%)	50.3 ± 1.7	51.9 ± 1.5	46.9 ± 2.0	**63.5 ± 1.4**
B → C Transition	TNEA	0.63 ± 0.04	0.64 ± 0.04	0.50 ± 0.05	**0.70 ± 0.03**
	SA (%)	48.9 ± 1.8	50.5 ± 1.6	45.5 ± 2.1	**61.2 ± 1.5**

**Table 7:** Performance under different spectrum overlap levels.

Spectrum Overlap	Metric	Baseline A	Baseline B	Baseline C	Ours
50%	TNEA	0.68 ± 0.03	0.69 ± 0.03	0.56 ± 0.04	**0.90 ± 0.01**
	SA (%)	55.2 ± 1.4	56.7 ± 1.3	49.8 ± 1.6	**86.5 ± 0.8**
40%	TNEA	0.75 ± 0.03	0.76 ± 0.02	0.63 ± 0.03	**0.93 ± 0.01**
	SA (%)	60.1 ± 1.3	61.7 ± 1.2	54.2 ± 1.5	**88.8 ± 0.8**
30%	TNEA	0.82 ± 0.02	0.83 ± 0.02	0.70 ± 0.03	**0.95 ± 0.02**
	SA (%)	66.9 ± 1.2	68.5 ± 1.1	60.2 ± 1.4	**90.6 ± 0.9**
20%	TNEA	0.89 ± 0.02	0.90 ± 0.02	0.78 ± 0.03	**0.97 ± 0.02**
	SA (%)	77.8 ± 1.2	79.3 ± 1.1	70.1 ± 1.4	**92.7 ± 0.9**
10%	TNEA	**0.97 ± 0.01**	**0.97 ± 0.01**	0.94 ± 0.02	0.95 ± 0.01
	SA (%)	93.0 ± 0.9	**93.6 ± 0.8**	91.5 ± 1.1	92.0 ± 0.8

**Table 8:** Sorting performance under varying SOLs.

SOL	2/16	4/16	6/16	8/16	10/16
TNEA	0.99 ± 0.01	0.98 ± 0.01	0.97 ± 0.01	0.96 ± 0.01	0.95 ± 0.01
SA (%)	99.2 ± 0.2	97.6 ± 0.4	95.9 ± 0.6	94.2 ± 0.8	92.3 ± 0.9

**Table 9:** Ablation study results.

Method Variant	SA (%)	TNEA
Proposed (Full)	92.3 ± 0.9	0.95 ± 0.01
w/o Multi-scale CWT (replace with STFT)	81.2 ± 1.71	0.75 ± 0.18
w/o KJDA Domain Alignment	76.8 ± 2.65	0.95± 0.01

## Data Availability

The data is unavailable due to some commercial issues.

## Abbreviations

**Symbol**	**Description**
*N*	Number of UAV targets
$\hat{N}$	Estimated number of UAV targets
*M*	Number of monitoring stations
$X_{m}$	Baseband signal
*L*	Total length of the baseband signal
$w_{m}$	Noise signal
$s_{i}(t)$	Baseband frequency hopping signal of the i-th UAV
$f_{i,k}$	Hopping frequency of the i-th UAV at the k-th dwell slot
$h_{m,i}$	Channel gain between the i-th UAV and the m-th monitoring station
$X_{m,\mathrm{pre}}$	Pretrained baseband signal
SPWVD	Smoothed Pseudo Wigner-Ville Distribution
$R_{zz}$	Correlation matrix of observation vectors
$a_{1},a_{2}$	Small and large scales of multi-scale CWT
W(a,b)	Continuous wavelet transform coefficient
$D_{s}$	Source-domain sample matrix
z	Target domain sample matrix
λ	Regularization coefficient of KJDA
σ	Width parameter of RBF kernel
**K**	Kernel matrix
$\tau_{i,m}$	Propagation delay from UAV i to station m
A	Optimal projection matrix obtained from KJDA
**Z**	Aligned feature matrix after KJDA projection
$r_{m}(t)$	Received composite signal at station m
$T_{h}$	Dwell time/hopping period
ε	CFAR detection threshold/SSP judgment threshold
TNEA	Target Number Estimation Accuracy
SA	Sorting Accuracy
SOL	Spectrum Occupancy Level
